# Mobile Phone Apps Targeting Medication Adherence: Quality Assessment and Content Analysis of User Reviews

**DOI:** 10.2196/11919

**Published:** 2019-01-31

**Authors:** Jamie Yea Eun Park, Jenny Li, Alyssa Howren, Nicole Wen Tsao, Mary De Vera

**Affiliations:** 1 Faculty of Pharmaceutical Sciences University of British Columbia Vancouver, BC Canada; 2 Collaboration for Outcomes Research and Evaluation Vancouver, BC Canada; 3 Arthritis Research Canada Vancouver, BC Canada

**Keywords:** medication adherence, mobile apps, mHealth

## Abstract

**Background:**

With the accessibility and widespread use of mobile phones, mobile phone apps targeting medication adherence may be useful tools to help patients take medications as prescribed.

**Objective:**

Our objectives were to (1) characterize and assess mobile phone medication adherence apps guided by a conceptual framework on the focus of adherence interventions and (2) conduct a content analysis of Web-based reviews to explore users’ perspectives and experiences with mobile phone medication adherence apps.

**Methods:**

We searched for mobile phone medication adherence apps using keyword searches in Apple and Android operating systems. We characterized all apps in terms of number of downloads, ratings, languages, cost, and disease target. We categorized apps according to 4 key features of (1) alerting to take medication, (2) tracking medication taking, (3) reminding to refill or indicating amount of medication left, and (4) storing medication information. We then selected representative apps from each operating system for detailed quality assessment and user testing. We also downloaded Web-based reviews for these selected apps and conducted a qualitative content analysis using an inductive approach involving steps of initial open coding, construction of categories, and abstraction into themes.

**Results:**

We identified 704 apps (443 from Apple and 261 from Android). The majority of apps across both operating systems had 1 or 2 features—specifically, 37.2% (165/443) and 38.1% (169/443) of Apple apps, respectively, and 41.4% (108/261) and 31.4% (108/261) of Android apps, respectively. Quality assessment and user testing of 20 selected apps revealed apps varied in quality and commonly focused on behavioral strategies to enhance medication adherence through alerts, reminders, and logs. A total of 1323 eligible Web-based reviews from these 20 selected apps were analyzed, and the following themes emerged: (1) features and functions appreciated by users, which included the ability to set up customized medication regimen details and reminders, monitor other health information (eg, vitals, supplements, and manage multiple people or pets), support health care visits (eg, having a list of medications and necessary health information in 1 app); (2) negative user experiences that captured technical difficulties (glitches, confusing app navigation, and poor interoperability), dosage schedule, and reminder setup inflexibility; and (3) desired functions and features related to optimization of information input, improvement of reminders, and upgrading app performance (better synchronization or backup of data and interoperability).

**Conclusions:**

A large number of mobile phone medication adherence apps are currently available. The majority of apps have features representing a behavioral approach to intervention. Findings of the content analysis offer mostly positive feedback as well as insights into current limitations and improvements that could be addressed in current and future medication adherence apps.

## Introduction

### Background

For many patients living with lifelong diseases, taking medications as prescribed is challenging. The World Health Organization has declared medication nonadherence as an epidemic and has called for feasible, patient-tailored solutions [1]. Particularly relevant, there has been surging interest in the use of mobile phones in public health practice (mobile health, mHealth) to address medication nonadherence, given their widespread use [2,3]. Indeed, mobile phones may represent a patient-centered means of targeting medication adherence, with features such as sending alerts to take medications, tracking doses, and supporting medication instructions.

A number of prior reviews have identified and described mobile phone medication adherence apps. In 2013, Dayer et al identified 160 apps on the Apple, Android, and Blackberry operating systems and subsequently published an update in 2017 with 645 apps to include those on the Windows operating system [4,5]. In 2014, Bailey et al extracted information on functions of 424 apps from app descriptions [3]. In 2016, Heldenbrand et al and Santo et al identified 347 and 272 apps, respectively, and categorized them based on author-identified features [6,7]. In 2017, Haase et al found 30 apps and classified ideal app features used to improve medication adherence [8]. In 2018, Ahmed et al analyzed 681 identified apps using app repository overviews or websites and mentioned the lack of health care professional involvement in the development of medication adherence apps [9]. These prior studies have incorporated various evaluation methods to assess app features, including using author-created rating systems [4,5,7,9], existing rating scales (eg, Mobile App Rating Scale and checklist for developing health literate mHealth apps endorsed by Institute of Medicine) [3,5,6,7,10], and user testing [4,5,9].

Although prior assessments of mobile phone medication adherence apps have added insight into these tools, they remain limited for various reasons, including evaluations based on app descriptions, short periods of trial, and user testing based on free versions. Furthermore, evaluations of app reviews have been limited [11,12]. Indeed, app reviews posted by the target users are publicly accessible and add to a valuable, naturally generated pool of data that to date have not been fully utilized. Altogether, the constantly growing number of mobile phone users [11] along with greater recognition of the problem of medication adherence in recent years [12] necessitates an update to aforementioned prior studies. In addition, an expansion of the knowledge on user experiences is needed.

### Objectives

As such, our objectives were to (1) characterize and assess mobile phone medication adherence apps guided by a conceptual framework on the focus of adherence interventions and (2) conduct a content analysis of Web-based reviews to explore users’ perspectives and experiences with mobile phone medication adherence apps.

## Methods

### Identification of Mobile Phone Medication Adherence Apps

We searched for mobile phone medication adherence apps on Apple (iTunes store) and Android (Google Play) operating systems during the month of May 2017. We applied 8 keywords (“medication,” “adherence,” “compliance,” “dose,” “dosing,” “drugs,” “reminder,” and “pills”) and did not impose inclusion criteria to ensure the broadest capture possible. However, apps were excluded if they were associated with services of specific pharmacies or businesses or had a primary purpose of advertising or other similar commercial activities. We downloaded each included app and extracted information on the operating system, number of downloads, rating, language, cost to download, and disease target.

### Characterizing and Assessing the Quality of Mobile Phone Medication Adherence Apps

To guide characterization of apps, we applied the conceptual framework on the theoretical targets of adherence interventions by assigning app features as *educational* (targets adherence by conveying information), *behavioral* (targets adherence by targeting, shaping or reinforcing specific behavior patterns), or *affective* (targets adherence through appeals to feelings and emotions or social relationships) [13,14] (see Multimedia Appendix 1). We then selected 10 representative apps from each operating system to conduct quality assessment and user testing. For Android, the primary selection criterion was number of downloads (≥100,000 downloads); in the event of a tie, the app with a higher average rating was selected. For Apple, as information on number of downloads is not provided, the selection criteria were on ratings followed by search retrieval order. Moreover, 2 authors who are clinically trained as pharmacists (JL and NWT) independently assessed the quality of selected apps using iPhone 5 (iOS9) and Samsung Galaxy Note 4 (Android Version 6.0.1) based on 12 features of [4] alerting to take medication; tracking medication taking (*behavioral*); reminding to refill or indicating amount of medication left (*behavioral*); storing medication information (*educational*); complex medication instructions or notes; database of medications; backup, cloud access, or means of access through another device; exportation or printing of data; free to download; alerts do not require internet connection; multiple profiles or patients; and multiple languages. In addition, ease of use was rated according to 3 levels: (1) easy—uses nontechnical language and involves functions that facilitate use (ie, drop-down menus) to minimize input, with usage of app usage learned in 5 min or less; (2) moderate—also involves functions that facilitate use but requiring greater degree of input, with usage of app learned in over 5 min but not less than 15 min; and (3) difficult—uses technical (medical or scientific) language, involves multiple functions, and requires substantial input, with usage of app learned in over 15 min. Authors discussed independently conducted quality assessments to come to a consensus for final reporting.

### Content Analysis of User Reviews

A qualitative content analysis was conducted on Web-based reviews for the 20 aforementioned apps. Specifically, we extracted user reviews submitted in English between January 1, 2017 and January 1, 2018 published on the official iOS app (Apple) and Google Play (Android) store and imported these into NVivo 11 (QSR International). We conducted a qualitative content analysis using an inductive approach and followed 3 main coding steps of (1) initial open coding, (2) construction of categories, and (3) abstraction into themes [15]. The constant comparative method was applied throughout the coding process [16]. We reached data saturation, a point of redundancy during the data analysis where no new concepts contributing to categories and themes arise [17], by the time the reviews for the twelfth app were coded.

## Results

### Identification and Quality Assessment of Mobile Phone Medication Adherence Apps

**Figure 1:**
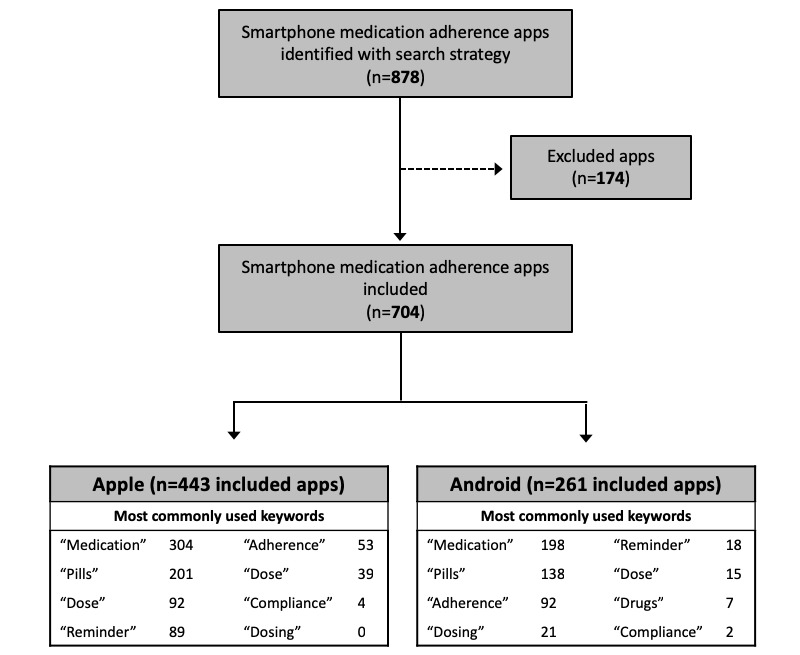
Flow of smartphone medication adherence apps included and most commonly used keywords (does not add up to number of apps since multiple keywords may be used to identify an app).

Our search strategy identified a total of 878 apps across both Apple and Android operating systems. After applying all exclusion criteria, 704 apps, with 443 from Apple and 261 from Android, were included (as shown in Figure 1).

**Figure 2:**
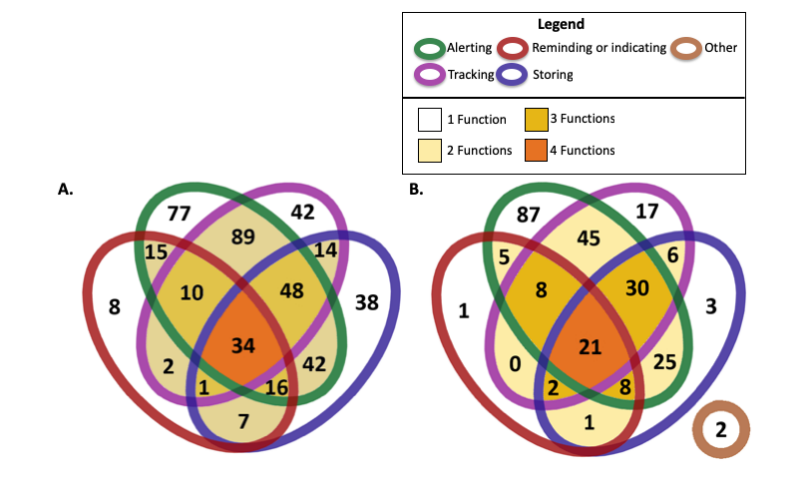
Smartphone medication adherence apps according to 4 key features: alerting (to take medication), tracking (medication taking), reminding (to refill)/indicating (amount of medication left) and storing (medication information) for A. Apple and B. Android operating system.

Table 1 summarizes the characteristics of included mobile phone medication adherence apps, including number of downloads, rating, language, and cost of downloading. The majority of apps across both operating systems had 1 or 2 features—specifically, 37.2% (165/443) and 38.1% (169/443) of Apple apps, respectively, and 41.4% (108/261) and 31.4% (108/261) of Android apps, respectively. Four-set Venn diagrams showing the possible combination of the 4 key features for included Apple and Android apps are shown in Figure 2.

**Table 1:** Characteristics of included mobile phone medication adherence apps.

Characteristics		Apple (N=443), n (%)	Android (N=261), n (%)
**Number of downloads^a^**			
	≤100 or unspecified	N/A^b^	83 (31.8)
	Approximately 500 to 1000	N/A	79 (30.3)
	Approximately 5000 to 10,000	N/A	66 (25.3)
	Approximately 50,000 to 100,000	N/A	20 (7.7)
	>100,000	N/A	13 (5.0)
**Ratings^c^**			
	≤1/5	0 (0.0)	4 (1.5)
	1/5<rating≤2/5	3 (0.7)	5 (1.9)
	2/3<rating≤3/5	0 (0.0)	17 (6.5)
	3/5<rating≤4/5	2 (0.5)	104 (4.0)
	4/5<rating≤5/5	7 (1.6)	98 (37.5)
	Unrated	431 (97.3)	39 (14.9)
**Languages^d^**			
	English	443 (100.0)	261 (100.0)
	German	83 (18.7)	0 (0.0)
	Spanish	83 (18.7)	0 (0.0)
	French	74 (16.7)	0 (0.0)
	Japanese	41 (9.3)	0 (0.0)
	Russian	40 (9.0)	0 (0.0)
	Simplified Chinese	37 (8.4)	0 (0.0)
	Traditional Chinese	24 (5.4)	0 (0.0)
**Cost of download (US$)^e^**			
	0.00	347 (78.3)	225 (86.2)
	0.00<cost≤1.00	2 (0.5)	4 (1.5)
	1.00<cost≤5.0	76 (17.2)	28 (10.7)
	cost>5.00	17 (3.8)	4 (1.5)
**Target**			
	General	328 (74.0)	200 (76.6)
	Contraceptives	32 (7.2)	35 (13.4)
	Asthma or chronic obstructive pulmonary disease	12 (2.7)	4 (1.5)
	Epilepsy	9 (2.0)	3 (1.1)
	Psychiatry	7 (1.6)	3 (1.1)
	Diabetes	3 (0.7)	3 (1.1)
	Other^f^	52 (11.7)	13 (5.0)
**Number of key features**			
	4	34 (7.7)	21 (8.0)
	3	75 (16.9)	48 (18.4)
	2	169 (38.1)	82 (31.4)
	1	165 (37.2)	108 (41.4)
	Other	0 (0.0)	2 (0.8)

^a^Numbers are approximated to “0”, “1”, or “5” in each digit.

^b^N/A: not applicable.

^c^Only apps that have ratings are included in this count.

^d^Each app can have multiple languages.

^e^Once the user begins using the app, the app may ask for additional costs not included in the cost of download.

^f^Other diseases included oncology, cardiology, and post-transplants.

With respect to apps with a single feature, the majority of which are involved in alerting, with 80 (17.9%, 80/443) in Apple and 87 (33.1%, 83/261) in Android. With respect to apps with dual features, the most predominant combination involved alerting and tracking with 89 (19.9%, 89/443) in Apple and 45 (17.1%, 45/261) in Android. The full list of mobile phone medication adherence apps identified is available in Multimedia Appendix 2.

Results of the detailed quality assessment with respect to additional features and user testing of 20 selected Apple and Android medication adherence apps are summarized in Table 2. For Apple apps, the 2 most common features (90%, 9/10) were alerting (to take medications) and alerts that do not require internet connection. With respect to user-friendliness, 40% (4/10) apps were rated as “easy,” 50% (5/10) as “moderate,” and only 10% (1/10) as “difficult.” Similarly, for Android apps, the 2 most common features (100%, 10/10) were alerting (to take medications) and alerts that do not require internet connection. Furthermore, all 10 Android apps assessed were free to download. With respect to user-friendliness, 20% (2/10) apps were rated as “easy,” 60% (6/10) as “moderate,” and 20% (2/10) as “difficult.”

### Content Analysis of User Reviews

User reviews were available for 14 (6 Apple and 8 Android) of the 20 selected apps, and altogether, 1323 reviews (331 Apple and 992 Android) were analyzed. Content analyses resulted in 3 themes: (1) features and functions appreciated by users, (2) negative user experiences, and (3) desired features and functions. These themes and associated categories are summarized in Table 3 and described in detail below.

#### Theme 1: Features and Functions Appreciated by Users

##### Category 1.1: App Performance and Practical Aspects

About one-third of the reviews in this category (34.16%, 452/1323) provided positive comments such as “Love this app!” (Android App #4) and “Awesome app” (Apple App #3) without specific details regarding which aspect of the apps were valued. For reviews that provided further details, user-friendliness and ease of use were commonly mentioned:

Would be good for all ages, including elderly people once set up [...] So far it seems excellent, I really like the format, it’s not made complicated by superfluous functions that never get used.

Android App #5

Some noted that they tried multiple apps before settling on their current app:

[...] I tried >8 till I settled on this one.

Apple App #3

Reviews also suggested frequent use of apps by users:

Use [the] app every single day...many times a day.

Apple App #2

**Table 2:** Quality assessment and user testing of selected mobile phone medication adherence apps in Apple and Android.

Features		Adherence intervention target	Apple (n=10 apps), n (%)	Android (n=10 apps), n (%)
**Assessment of quality according to availability of features**				
	1. Alerting to take medication	Behavioral	9 (90)	10 (100)
	2. Tracking medication taking	Behavioral	7 (70)	7 (70)
	3. Reminding to refill or indication amount of medication left	Behavioral	5 (50)	7 (70)
	4. Storing medication information	Educational	8 (80)	7 (70)
	5. Complex medication instructions and/or notes	Educational	6 (60)	8 (80)
	6. Database of medications	Educational	5 (50)	2 (20)
	7. Backup, cloud access, or means of access through another device	N/A^a^	7 (70)	6 (60)
	8. Exportation or printing of data	N/A	6 (60)	5 (50)
	9. Free to download	N/A	8 (80)	10 (10)
	10. Alerts do not require internet connection	N/A	9 (90)	10 (10)
	11. Multiple profiles or patients	N/A	5 (50)	5 (50)
	12. Multiple languages	N/A	3 (30)	2 (20)
Number of features, mean (range)		N/A	6.5 (3-9)	6.6 (2-10)
**Assessment of user friendliness**				
	Easy	N/A	4 (40)	2 (20)
	Moderate	N/A	5 (50)	6 (60)
	Difficult	N/A	1 (10)	2 (20)

^a^N/A: not applicable.

**Table 3:** Themes and categories emerging from content analysis of Web-based reviews for selected mobile phone medication adherence apps (N=1323 user reviews).

Themes and categories		Reviews, n (%)^a^
**1. Features and functions appreciated by users**		
	1.1 App performance and practical aspects	765 (57.82)
	1.2 Helpful reminders and notifications	540 (40.81)
	1.3 Monitoring other health information	80 (6.05)
	1.4 Versatility of medication information input and display	79 (6.04)
	1.5 Supports health care visits	63 (4.76)
**2. Negative user experiences**		
	2.1 Technical difficulties	393 (29.70)
	2.2 Challenges with medication information input and display	58 (4.38)
	2.3 Problems with reminders and notifications	40 (3.02)
**3. Desired functions and features**		
	3.1 Optimizing medication information display and input	73 (5.51)
	3.2 Improving reminders and notifications	39 (2.94)
	3.3 Upgrading app performance	25 (1.88)

^a^The percentages of the user reviews coded do not add up to 100% because user reviews can be coded to multiple categories or themes.

In addition, recurring positive comments referred to the willingness of users to pay for the pro-version of the app for desired features and functions:

Highly recommended, even if you take only one medication or vitamin a day. Worth paying for the upgrade to Premium version to get all the customize options - especially for those who need a visual of individual medications.

Android App #5

Users also appreciated the user interface being “elegant in a friendly minimalistic way” (Apple App #3). Commonly, reviews complimented the apps as having a “clean and simple format” (Apple App #3) and a “beautiful layout” (Apple App #2). Other practical aspects of the apps that users enjoyed include no advertisements, doing what the app says on its description, providing friendly and swift customer support, and constant upgrades to the app to fix existing problems and add new features.

##### Category 1.2: Helpful Reminders and Notifications

Around 29.47% (390/1323) of the reviews expressed that reminders supported users with remembering and keeping track of their medications. For users who recently got diagnosed with a condition and/or recently started to take a handful of medications, they found the reminder features valuable:

I recently had a medical issue that required me to be on several medications.I've never taken meds regularly and this app helped me make sure I took everything when I was supposed to. Highly recommend.

Android App #6

Frequently, reviews mentioned the benefits of setting reminders not only for the users’ medications but also for refills:

Reminds me when to take it. Reminds me went to refill the prescription. Absolutely essential to those who take medication.

Apple App #2

Other aspects of helpful reminders and notifications include ability to customize alarm sounds and snooze feature to defer taking medications. For example:

I love that you can choose what alarm tone you can use. I have to take two medications and I am able to set two different tones.

Android App #3

##### Category 1.3: Monitoring Other Health Information

Alongside managing prescribed medications, apps helped with monitoring other health information such as nonprescription medications (eg, supplements), vital measurements (eg, blood pressure, blood sugar, and pulse), symptoms (eg, pain), and drug safety (eg, side effects). Other less commonly mentioned health information that users monitored with their apps included bowel movements, cigarette usage, diet, exercise, and water consumption. Many reviews praised the ability to manage multiple people or pets. Furthermore, 1 user mentioned the following:

Now I love the multiple patients feature [my dogs have no phones to track their meds :)], and it is quick to switch from one to another.

Android App #5

##### Category 1.4: Versatility of Medication Information Input and Display

Many reviews were complimentary of the customization of the medication input. These included the ability to embed medication details (eg, adding shape or color or type of medications, and inputting notes or pictures) and add personalized dosing options (eg, varying frequency settings and scheduling dosage change). The apps were able to accommodate unconventional dosing schedules such as medications that users take once a week, alternative weekend hours, and every 2 weeks. In terms of medication input display, many users commented on the useful features to track inventory, “help [users] keep track of all of what and when [users] have taken [their] pills” (Apple App #3) and generate summary reports to “produce a list of current medications and to graph my progress of selected vitals” (Android App #4).

##### Category 1.5: Supports Health Care Visits

Reviews frequently expressed the convenience of having their list of medications and necessary health information in 1 app. For instance:

This is an outstanding app. It's so helpful when going to the doctor. I just show them my phone, no more dragging pill bottles with me! So much you can do with this app. I love it!

Android App #2

Users mentioned a wide range of occasions when the app is handy including single or different health care providers visits, emergency rooms, and for self-medication management. In particular, users found keeping track of health care visit appointments and linking prescriptions to the corresponding physician or pharmacy helpful.

#### Theme 2: Negative User Experiences

##### Category 2.1: Technical Difficulties

Around 15.79% (209/1323) of the negative user experiences related to technical glitches and bugs from the apps that would disrupt the app features and functions, particularly the reminder feature (eg, not notifying user at the correct time and app crashes). Recurring complaints also referred to difficulty and confusion in app setup and use:

There is no tutorial or help section on the app, so you just blindly have to click on things to try to figure out how to set anything up for the first time.

Apple App #4

Reviews also commented on the “clunky” user interface (app visual design):

Popup menus have dark text on dark background, so they can't really be read.

Android App #4

Very slow menus and animations for no reasons, lots of extra buttons and sub menus.

Android App #3

Poor interoperability was another area that users noted. Users mentioned inadequate synchronization and backup with multiple devices and programs: inability to transfer data to Secure Digital card, to sync between multiple devices, and the lack of “website database or cloud to restore database files from” (Android App #4). Particularly, for Apple watch, reviews mentioned the issue of reliability:

It links with Apple Watch but does not sink back with your iPhone. So if you click on 'take' it does not register with the iPhone app and thus keeps pinging you to take your meds.

Apple App #2

Users also referred to unsatisfactory customer service and updates. Many users mentioned they “submitted many feedback messages about [a] problem and no response or fixes [were made]” (Android App #5). Users commonly experienced long waits, with no response or outdated, unhelpful response from customer support. Regarding app updates, some reviews noted that there have not been any recent updates or that the new updates cause more problems (eg, glitches, more advertisements, and more confusing). For users that pay a subscription fee, reviews similarly criticized:

I really like this app, however there appears to not have been any updates since April. If there are to be no improvements should you be charging? And especially at the price you are asking?

Android App #5

Other technical difficulties identified in reviews were cost (eg, expensive and necessity of subscription of pro version to use the app), high volume of advertisements, burden on the device (eg, large device storage and high use of battery and data), discrepancy with app description and actual app features, and lack of security measures (eg, user confidentiality).

##### Category 2.2: Challenges With Medication Information Input and Display

Inflexible information input was a recurring complaint which consisted of difficulty inputting medications from different countries because of “inadequate medicines information base” (Apple App #2) and inability to customize dosing schedule (eg, scheduling different dosing schedule based on the day). Users often have personalized drug regimens that they need to adhere to, and they expressed that several apps do not reflect their or their loved one’s correct drug regimen, for example:

The only issue that I didn’t like was I have 1 medicine that I only take Sun-Thur and I don’t have that option [...].

Android App #6

This problem was also applicable to special populations such as children because “[…] children's dosages are determined by weight so sometimes you get weird doses for customized medications.” (Apple App #5).

Users mentioned the inability to “[…] update the time of the subsequent dosages throughout the day” (Android App #3) based on the time users take the medication, which may lead to taking medications at improper times. Inability to input supplementary notes or pictures (eg, adding “whether to take meds with food, or before or after meals” [Android App #3]) and fix mistakes in medications logs were challenges that users also expressed. In terms of medication information display, reviews referred to the inconsistency of units, unfavorable display in military time, inability “to view a list of dates and times meds were taken” (Android App #3), and inability to view and track balance of medications remaining. An example of a user review includes:

Was great until I tried added an oral suspension medicine: the app would only allow me to enter amounts in grams, instead of ml. I don’t see a user-friendly way of choosing a unit.

Apple App #5

##### Category 2.3: Problems With Reminders and Notifications

Users most commonly expressed challenges with limited alert customization, especially in terms of the alarm loudness. Specifically, users commented that at night, “no matter what tone, the tone is too soft and doesn’t ring long enough to pull me out of my sleep” (Android App #6). Users mentioned that the customization with ringtone is “a very important feature, since it is how you will be notified” (Android App #6). Some complaints evolved around the inability to prioritize reminders if multiple medications are taken at the same time:

[...] if you take 6 meds at 13:00, you'll get 6 notifications at the same time, sounds like your phone is having a seizure.

Android App #3

For 1 app in particular, users noted their frustration on the app’s incapability to adjust the schedule based on the Daylight Savings Time: For users who “[...] take several medications every day, and is really a pain to have to go in and change the time on each one” (Android App #5). Some reviewers mentioned the hassle of unlocking their phones for their alarms to ring or to record as taken, for example, “USELESS. No alarm unless u open app!” [Android App #5]

#### Theme 3: Desired Functions and Features

##### Category 3.1: Optimizing Medication Information Display and Input

Flexibility of data input was a request that commonly appeared. Users wished for sections to add notes for details on their specific medication or dose, their symptoms, or mood. They also requested to add pictures of their medication bottles and pills. Frequently, users mentioned that “dose options need to reflect actual doses” (Apple App #2), particularly in terms of being able to schedule different dosing schedules and dosage change based on the day, for example:

Some prescriptions have you take one pill one day and two on another. Therefore, it would be good if you could set it to a different number of pills on specific days. I know I could just add the prescription more than once, but then tracking the number of pills remaining wouldn't be accurate.

Apple App #2

Many users desired to have the ability to readjust their drug schedule based on the actual time the drug was taken:

Would be nice if you could define a dose to be given X hours after the previous one, instead of strictly every X hours, in case a dose was given late.

Android App #4

Fewer user reviews requested for the ability to add 0.5 portions and to have a more adjustable dosing frequency, barcoding or scanning function to easily input their medications, and unit setting. Improvements to the medication history section include being able to “summarize medication activity” (Android app #3) by having “an option to enter end date” (Android App #6) and by being “able to look back at the actual times when a medication dose is taken, not just that it was taken.” (Android App #3).

##### Category 3.2: Improving Reminders and Notifications

Users mainly made requests on 2 particular aspects of reminders: customization of reminder setups and suggestions on new, beneficial features. Reviewers asked for alert customizations in terms of loudness and ringtones, reminder time frame, and involvement of their caregivers or family members in their care. Users also requested for more efficient methods to indicate medications as taken without opening the app. For example, a user specifically suggested “I only wish I could use voice command to ‘take’ medicine in the middle of the night when in pain without fumbling for my glasses.” (Apple App #3).

##### Category 3.3: Upgrading App Performance

Better interoperability was the request that most frequently appeared in this category. Users wished for enhanced linkage to other devices (eg, Apple Watch) and programs (eg, Web version of the app, other Apple or Android devices, and Dropbox) to sync or manage their data and appointments. As 1 user summarized:

Synchronization between 2 devices, [in] other words, [require] the app [to] run on 2 devices and something entered on one device can also be seen on another one (i.e. phone and iPad).

Apple App #2

## Discussion

### Principal Findings

This study provides better understanding of medication adherence apps from dual perspectives. First, quality assessment and testing by pharmacist researchers provides a health care provider’s lens, and second, content analysis of reviews provides a target user’s lens. Major strengths of this study include an update to the current landscape of 704 medication adherence apps. The subsequent content analysis of user reviews conducted soon after the identification and quality assessment of apps adds uniqueness to our study. The apps analyzed were still available in their corresponding app stores and allowed the authors to compare results of our quality assessment. Indeed, although qualitative analyses of user reviews for disease-specific apps including for bipolar disorder and weight loss [18,19] have been previously published, target users’ (patients’) experiences with medication adherence apps have not been extensively studied. Previously, Stawarz et al in 2014 conducted a user review analysis of the top 50 reviews for 40 apps available only on the Android operating system [20]. Bailey et al in 2014 conducted a user review analysis of 26 eligible apps that appeared in their initial search results [3]. However, these were limited to an arbitrarily chosen top 75 “most helpful” reviews, and imposing preidentified themes to their analysis made it largely deductive instead of allowing themes to be inductively generated from the data. Therefore, our systematic strategy to identifying apps, replicable steps (eg, based on dates) for selecting reviews, and purposeful application of content analysis methodology provides a more in-depth, rigorous approach to understanding users’ experiences and perspectives with mobile phone medication adherence apps.

Indeed, combining quality assessment and user testing of apps with qualitative analyses of corresponding Web-based reviews provided the opportunity to contextualize respective findings. For example, user reviews were mostly positive, and the main theme that emerged was features and functions appreciated by users. We noticed that the more commonly a feature was available (eg, alerting), the higher the number of user reviews were present in their corresponding category (eg, reminders and notifications). Most users found the reminders and notifications features helpful for multiple medications including nonprescription or as-needed medications. Users generally found apps to be user-friendly and appreciated the simple app design. These characteristics address existing barriers of difficult app navigation and the time consumption when inputting their medications [21,22]. Despite the previously expressed challenges, including the inability to create reminders without internet connection and for multiple people on numerous medications [21], our results reveal that currently, the majority of the apps (90% [9/10] for Apple and 100% [10/10] for Android) do not require internet connection and include the ability to manage medications for multiple people and pets.

A practical finding from our study is that users commonly expressed that they tried multiple apps before settling on one that they favored. Moreover, one of the reasons that could be associated with this frustration may involve search terms [21,23]. The quantity of adherence apps yielded by the search results varied significantly among keywords. The search terms “adherence” and “compliance” yielded relatively few relevant search results on both operating systems (53 and 4 apps on Apple and 39 and 2 apps on Android). On the contrary, the terms “medication” and “pills” yielded the most results on both operating systems (304 and 201 on Apple and 198 and 138 on Android). This reveals preferences the public, or at least the technology community, may have with regard to the language that is used to discuss the topic of medication adherence. Patient-friendly terms (eg, “pills”) appear more frequently used than relatively jargon-like terms used by the medical community (eg, “compliance”). Health care providers who may be recommending these apps to patients may benefit from being aware of the types of language and terminology preferred by the specific patients they are caring for.

### Limitations

During the app identification process, only single search terms were used. It is not known whether the usage of compound search terms would have yielded a larger number of results or perhaps more tailored results. We extracted user reviews for content analysis within the 1-year period (January 1, 2017, to January 1, 2018), and since then, there may be different versions of the apps that may have appeared or removed. Furthermore, given the sheer number of available apps, we limited content analyses to representative apps from each operating system and only those submitted in English, as such reviews may not accurately represent the entire population of app users. In addition, individuals providing reviews may be systematically different from those who do not—in that they are likely those who strongly favor or dislike apps.

### Conclusions

Our app quality assessment and content analysis of user review study provide a view of the available mobile apps for medication adherence and the target users’ (patients’) experiences with medication adherence mobile apps. Our findings can inform the future development of the next generation of medication adherence apps co-designed with patients, researchers, and technology companies.

## Appendix

Multimedia Appendix 1 Explanation of app features and application to conceptual framework.

Multimedia Appendix 2 Complete list of medication adherence apps identified.

## Abbreviations

mHealth: mobile health
