# Supplementary material for: Mobile Phone Apps Targeting Medication Adherence: Quality Assessment and Content Analysis of User Reviews
Source: JMIR Mhealth Uhealth. 2019 Jan 31;7(1):e11919. doi: 10.2196/11919 (PMC6374723; doi:10.2196/11919)
Supplement: Multimedia Appendix 1 [file mhealth_v7i1e11919_app1.pdf]

Appendix 1: Explanation of app features & application of conceptual framework.

App Features	Adherence Intervention Target	Explanation
**Alerting (to take medication)**	Behavioural	Patients first input information about when and how the medication is to be taken, which then allows the app to initiate a reminder/alarm to the patient at the intended time that it is time to take their medication.
**Tracking (medication taking)**	Behavioural	Ability for patients to input instances that they have taken their medication(s) into the app, thus providing the opportunity to track execution of the dosing regimen.
**Reminding (to refill)/ Indicating (amount of medication left)**	Behavioural	Patients first input information about when and how the medication is to be taken, which then allows the app to initiate a reminder to the patient at the intended time. Reminding to refill may be an alert/alarm that reminds patients that it is time to obtain a refill of their medication. Alternatively, this may also be an alert that indicates to patients how many/much medications they have left, thus signaling the need to refill.
**Storing (medication information)**	Educational	Capability to capture and view patients' list of medications in a way that clearly displays the name of the medications and other regimen-related information (e.g., strength, dosage, frequency) that patients could then present to their physicians as a medication history or to their pharmacists as part of a medication review. While this feature has elements of behavioural, educational and affective, this was assigned as an *educational* feature as it largely conveyed information.
