# Supplementary material for: Mobile Phone Apps Targeting Medication Adherence: Quality Assessment and Content Analysis of User Reviews
Source: JMIR Mhealth Uhealth. 2019 Jan 31;7(1):e11919. doi: 10.2196/11919 (PMC6374723; doi:10.2196/11919)
Supplement: Multimedia Appendix 2 [file mhealth_v7i1e11919_app2.pdf]

Appendix 2: Complete list of medication adherence apps identified.

Operating System	App Name	Developer
**Apple**	Pill Monitor<br>*(Assessed for quality, analyzed user-reviews- Apple App #1)*	Maxwell Software
	Medisafe Pill Reminder, RX & Medications<br>*(Assessed for quality, analyzed user-reviews- Apple App #2)*	MediSafe Inc.
	Round Health<br>*(Assessed for quality, analyzed user-reviews- Apple App #3)*	Circadian Design
	MedOClock<br>*(Assessed for quality, analyzed user-reviews- Apple App #4)*	M2C2 COMMUNICATIONS INC.
	DrugHub: Medicine cabinet in your pocket<br>*(Assessed for quality, analyzed user-reviews- Apple App #5)*	The Great-West Life Assurance Company
	MeAndMyPharmacist.ca<br>*(Assessed for quality, analyzed user-reviews- Apple App #6)*	L'Association Quebecoise Des Pharmaciens Proprietaires
	iHealthTrax Free<br>*(Assessed for quality)*	Winkpass Creations, Inc.
	iMediLog<br>*(Assessed for quality)*	Flexicoder Ltd,
	Pill Monitor Pro - Medication Reminder<br>*(Assessed for quality)*	Maxwell Software
	Pill Reminder Pro (Push Notification)<br>*(Assessed for quality)*	Winkpass Creations, Inc.
	21 - Birth Control Pills Reminder	Ernesto Fernandez
	A1chronicle	Kairos Development Co
	AAP Asthma Tracker for Adolescents	American Academy of Pediatrics
	Accredo Plus C	Accredo Health Group, Inc.
	ACMC	GenieMD, LLC
	ADHD Health Storylines	Self Care Catalysts Inc
	Adherence Project	Deemsys Inc
	Adherence to AHT	Telybnova Research
	After Meal Checklist	Masaki Kanno
	AppaGrapha	Odin Pharmacy Innovations
	Asthma Storylines	Self Care Catalysts Inc
	AsthmaCheck	axovis GmbH

AvisaMED!	SaludMOVIL.net
Axilla PHR	CodeMonkee d.o.o.
BC Pill Pro	Sebastian Lang
BC Pill Reminder	Sebastian Lang
Besider Birth Control Reminders	Bedsider LLC
Birth Control Reminder	Sergio Licea
Birth Control Reminder myPill	Bouqt.com Ltd
BladderTrakHer	American Urogynecologic Society
BonaPill Contraceptive Reminder	ALFA MEDIMEDIA, s.r.o.
BP Wiz	LINKLINKS LTD
BP Wiz Pro	LINKLINKS LTD
Capzule PHR	Webahn, Inc.
Cardiomyopathy	BLUEFIGMENT LIMITED
CardioSmart Med Reminder	American College of Cardiology
Care Control Medication	Care Control Systems Ltd
Care Log	Tung Tree LLC
CareFlow PHR	Brian Biddulph-Krentar
CareSync	Caresync, Inc.
CareZone	CareZone
CeyHello FREE	MedSqr LLC
CF MedCare	M-Data SA
Chemo Aid	Edward Dixon
Circle	Onur Yoeruek
CircleCare	CircleCare, Inc
Claris for Caregivers	Claris Healthcare
CML Today	Leukemia Patient Advocates
Contraceptive Ring Reminder	Rodrigo Corbelli
Contraceptive Ring Reminder	Philipp Blanke
Cycle Reminder	Target Work Servicos de Informatica Ltda - ME
Daily Medicine	Am mA
Daily Pill	Maciej Gorecki
Dawaai	MAG Software

	DBSA Wellness Tracker	Depression and Bipolar Support Alliance
	denovoMD	Denovo Health LLC.
	Diabetic Organizer On Go	WizenX Software Solutions Private Limited
	dialogMS	Merck KGaA
	Do not forget your pills	MakeItApp
	Don't Forget Remedy	Health Preference, LLC
	Don't forget Remedy GOLD	Health Preference, LLC
	Dose Anywhere	DOSE Health
	Dose Direct: medication, pill reminders	Omi Studio
	DoseBox - Medication Diary	DigitalStitch Limited
	Dosecast	Montuno Software, LLC
	DoseMe	Luna Software
	DoseOrganizer	Log n Labs LLC
	DoseOrganizer Lite	Log n Labs LLC
	DoseSmart	DoseSmart
	Dr. Heimer	Enrique Galicia
	Drug alert everyday	Alongkorn Khantawong
	Drug Expiry	MakeItApp
	Easy Pill	BirdsCorp.com
	ELFLy Asthma	Joe Robertson
	ELFy Epilepsy	Joe Robertson
	emocha miDOT	emocha Mobile Health
	emWellics	RxEOB
	EncourageApp	Anil Thomas
	e-Nurse	HIROFUMI MARUO
	Epi & Me 2	HandMe
	EpiDiary Australia	Irody, Inc.
	Epilepsy Foundation My Seizure Diary	Epilepsy Foundation of America
	Epilepsy Health Storylines	Self Care Catalysts Inc
	Epilepsy Ireland: Epilepsy Management	Epilepsy Ireland
	ePills	JAVIER LLAVERIA
	Epilpsy Tool Kit	Epilepsy Society

	Every Dose, Every Day	Centers For Disease Control
	Family Med Track	Alexander Software
	Family Medical Manager	Acadian Companies
	FamilyRx Tracker	Renaud Holcombe
	FamilyRx Tracker	Paul Grotefend
	FBCompliance Reminder	NIJIATI ABUDUKAIYOUMU
	Flo Period Tracker	OWHEALTH, INC.
	Florence	Nerve AB
	Furiend	MCRUNCHER R&D SDN BHD
	GenieMD	GenieMD, LLC
	GenieMD + OH	GenieMD, LLC
	GoodRx	GoodRx
	HabiTimer	Arciem LLC
	Hart	Hart, Inc.
	Headache Diary (ecoHeadache)	ecoTouchMedia.com
	Headache Diary App	Furia 7
	Health Log Keeper	A1Brains Infotech
	Health Mapper	Prosper
	Health Pocket	RATNAKALA SOFTWARE PRIVATE LIMITED
	Health Pro	Adeline Labs Inc.
	Health Reminder	Lin Li
	Health Reminder Pro	Lin Li
	Health Storylines	Self Care Catalysts Inc
	Health Tracking from Doctor Care Anywhere	Doctor Care Anywhere Ltd.
	HealthAssist	WellCare Today, LLC
	HealtheMinder	Hartford HealthCare
	HealthFlo	Milan Patel
	HealthHelm	CertiCon a.s.
	Healthnote	Health Enterprises
	HealthSavvi	HealthSavvi Inc
	HealthTouch	HealthTouch

	Health-Track	Syed Abdul
	HealthyWatch	Intelligent Decisions
	HeartHealth	Giacomo Ferrari
	HexCare	HexCare
	iAwaken	Green Steps Ahead, LLC
	IBM HealthCoordinator	GenieMD, LLC
	iCANcer	Naomi R. Bartley
	iCancerHealth	MEDOCITY, INC.
	iCare - Medication Reminder	iDaily Corp.
	iCARE Clinic	Madhukar Varshney
	iHeadache	BetterQOL.com
	iHealth Log	Diffraction Limited Design LLC
	iHealthTrax	Winkpass Creations, Inc.
	iMED Tracker	Simplified APP Solutions, LLC
	iMeds - Pill and Medical Appointments	iDevMobile Tec.
	iMigraine	Softarch Technologies AS
	Infusion Express	Play-it Health
	Inhale	Skyhawk Media LLC
	Inhaler Counter App	Jules Gribble
	Inhaler Tracker	Shaun Austin Limited
	Injection Tracker	AbbVie
	iPatientCare	iPatientCare, Inc.
	iPharmacy - Pill ID & Rx Reminder	SigmaPhone LLC
	iPill	Soontorn Auksorncherdchoo
	i-pill	Mambo S.A.
	iPills - Medication Reminder	Appgorithm, LLC
	iRxHelper	JAZ Software Solutions
	iRxHelper Lite	JAZ Software Solutions
	iRxManager	CommandSoft, Inc.
	i-SaiSo Wellbeing Monitor	i-SaiSo
	iStayHealthy	Peter Schmidt
	iTake Mobile Medication List	Andrew Slavetskas

	iTake: Supplement and Pill Tracker	Dima Bart
	Jill's Pills	Alaska App Works
	KeepRx	Kiawe Tech, LLC
	Kids Cancer Meds	Ddavid Ziegler
	Kid's Health Tracker	Chris Nielsen
	Lady Pill Reminder	Bouqt.com Ltd
	Liekovka & Pill reminder	Webster, spol. S r.o.
	Lima Memorial Health System	Axial Exchange, Inc.
	ListMeds	Control Catalyst Inc.
	Lupus Corner Health Manager	Karate Health, Inc
	Med Alert	Horia Beschea P.F.A
	Med Helper	Earth Flare, Inc.
	Med Reminder	Fortyfox
	Med Time	Eduardo Halfen
	Med Timers	Douglas Dyer
	Med Track Lite	Ceri Turner
	MedaCheck Habit	MedaCheck LLC
	MedApp	PharmIT
	MedBox	DogTown Media, LLC
	MedCompanion	Pedro Ramon Ventura Gomez
	MedControl	Rambletta Ltd.
	Medfy	MedfyOnline.com
	medHilfe	I-Motion GmbH
	MedHistory	DocSoft Apps
	medi reminder	Frederik Frandsen
	MediAlert	Seasia Infotech
	Medic Owl	CuatroD
	Medicaion Passport	Imperial College Healthcare NHS Trust
	Medical Diary - All in ONE	YangYeon Cho
	Medical Reminder	RATNAKALA SOFTWARE PRIVATE LIMITED
	Medicate	Elliott Minns
	Medicate	Christopher King

Medication Adherence	Right to Care
Medication Alarm	new media company GmbH & Co.KG
Medication call reminder	TeleStar LTD
Medication Care Card	AppDesk Solutions
Medication Diary and Drug List	Clarity Health Inc.
Medication Dose Log	Manu Gupta
Medication Genie	Shiv Verma
Medication in order	Kirill Flippov
Medication Log	Manu Gupta
Medication Manager On Go	WizGenX Software Solutions Private Limited
Medication Passport	Imperial College Healthcare NHS Trust
Medication Reminder & Pill Tracker	smartpatient GmbH
Medication Tracker (iMedications)	iHealth Ventures LL.
Medicine Manager	Elliot Barer
Medicine Reminder	Paulo Correia
Medicine Reminder HD	Pop-ok.com
Medicine Reminder HD - with Local	Pop-ok.com
Medicine Reminder Pro	Jay Shenmare
Medicine Tracker	Ceri Turner
MedicineAlert	Roel Lakenvelt
MedicineManage	zhuxian zhu
Medicines Expiry Reminder Lite	Janarthanan V
Mediclock	Media Marketing Lab
MediKite	Leon van de Broek
Medimate	Doublewedge
Medi-Prompt - Medication Reminder	Thornsdale
MediRecords	MediRecords Pty Ltd
MEDI-SCAN	www.PulseSolutions.com
MediWare	Jagadish Sudarsanam
Medkit	Oleksandr Zelenyi
MedKit Lite	Oleksandr Zelenyi
MedMemo	INLABS Sp. z o.o.

	MedMinder - University of Iowa	The University of Iowa (ITS)
	Medmonitor	Benesalus Technologies LLC
	Medocity COPD Care	MEDOCITY, INC.
	Medopad Patient Monitoring	Medopad Ltd
	MedOptimizer ADD/ADHD for Adults	Hunt- Psychiatric Innovations
	MedOptimizer Depression	Hunt- Psychiatric Innovations
	MedOptimizer OCD	Hunt- Psychiatric Innovations
	MedOrganizer	PanHealth Inc.
	MedOrgMarathi	PanHealth Inc.
	MedRemind	Jay Lagare
	Med-Reminder Patient Access	MedM Inc
	Meds & Pills Reminder	Dr. Cuco
	Meds Agenda	DIGI117 LTD
	Meds Tracker: Medication Daily Reminder	Leonid Lemesev
	MedsConnect Medicine Reminder	Amira Awwad
	MedsDiary	Mario Iannotta
	MedsListLite	HYBSW
	MedsMinders	TECHtionary.com
	MedsMinders Unlimited	TECHtionary.com
	MedsMinders+3	TECHtionary.com
	MedsMinders+5	TECHtionary.com
	Meds-reminder	Stefan Volker
	Medtep Asthma	Medtep Inc.
	MedTracker	Fairwinds Software
	Medtrek	johannes mhembere
	MedXCom for Patients	Giffen Solutions, Inc.
	Medyc	Mazen Kourouoche
	Medy-Sweeten the pill	Vaica
	Mega Meds	AppAttic Ltd
	Memo Health	Tinylogics Ltd

	MeMyHealthAndi	JAZ Software Solutions
	Mhi-Go	Johns Hopkins Digital
	mICE Health	Stealth Productions Limited
	Migraine Buddy	Healint
	Mr. Pillster	Whisper Arts
	MS Management	Emily Whisker
	mSmart Medication Aide	Intelligent Automation, Inc
	My Asthma Manager	Point of Care
	My Bladder Cancer Manager	Point of Care
	My Breast Cancer (BC) Patient Companion	Point of Care
	My Cardiac Coach	American Heart Association
	My Chronic Lymphocytic Leukemia Manager	Point of Care
	My COPD Manager	Point of Care
	My Cystic Fibrosis Manager	Point of Care
	My Diabetes Home	My Diabetes Home, LLC
	My Diabetes Manager	Point of Care
	My Doctus	Pixentia Corporation
	My Fight Against Asthma	Brian King
	My Fight Against Cystic Fibrosis	Brian King
	My GIOTRIF	Boehringer Ingelheim Pharma GmbH & Co.KG
	My Hypoparathyroidism Manager	Point of Care
	My IPF Manager	Point of Care
	My Major Depressive Disorder (MDD) Manager	Point of Care
	My Medical	Hyrax Inc.
	My Medical Info	Deardorff Digital LLC
	My Medication	Asan Medical Center
	My Medication Diary	Baskaran Arunasalam
	My Medication Journal	Ara Wolf
	My Medicine Box Kit	Kandimalla Puspavati
	My Multiple Myeloma Manager	Point of Care
	My NSCLC Manager	Point of Care

	My OC	Consilient Health Ltd.
	My OC Diary	Toroku Shimizu
	My OC IRL	Consilient Health Ltd.
	My Osteoporosis Manager	Point of Care
	My Personal Health	www.ajaxmediatech.com
	My Pill	LePro
	My Pill	Kaeli Soft
	My PillMinder	Alvaro Ruiz
	My Prostate Cancer Manager	Point of Care
	My Renal Cell Carcinoma Manager	Point of Care
	My Rheumatoid Arthritis (RA) Manager	Point of Care
	My Skin Cancer Manager	Point of Care
	My Treatment	Connectmedica Sp. Z o.o.
	my uBox	Abiogenix Inc.
	Mycrisanta	Cipla Ltd
	myGIST Companisn	Novatis Pharmaceuticals Corporation
	myIBD	The Hospital for Sick Children
	myLDN	LDN Research Trust
	MyMate&Me	Condat AG
	MyMedimate	Compubits Solutions Pvt.Ltd
	MyMeds	MyMeds, Inc.
	MyMeds Medication Reminder	Alixandra Price
	MyMedsApp	Kathryn Robertson
	MyMobilePharmacist	Ohio State University
	MyReminder	Aspyre Solutions
	myRx Planner	Blue Cross and Blue Shield of Alabama
	MyRxList	David Bellerive Lamarre
	MyRxTracking	EagleForce Associates, Inc
	MyVitals	Lazy Dog Utilities
	NUH myMeds	National University Hospital (Singapore) Ptd Ltd

	NuvaRing Reminder App	Merck & Co Inc
	Oculus Health	Sridhar Yerramreddy
	On My Pillbox	Alex He
	OnTablets	masaki individual
	OntheGoRx	Ateb, Inc.
	OnTimeRx FREE	AmeliaPlex, Inc
	OnTimeRx PRO	AmeliaPlex, Inc
	Oobat - medicine reminder	iReka Soft
	OpenMe Cap	Bobby Mestepey
	Pain Diary	Sanovation AG
	PastiMed Lite	Mediaexpander
	PastiMed Pro	Mediaexpander
	Patient Diary	Antix Software Limited
	Pet Mediation	Manu Gupta
	PetMinderApp	TangoSquared
	Pets Tracker	LINKLINKS LTD
	Pharmasave Drugs	Pharmasave
	Piill 4 Me	Hormosan
	Pill	Marco Tini
	Pill Alert	SIMPTEK LLP
	Pill Alert	Jaladi Corp
	Pill Alert	LINKLINKS LTD
	Pill Alert - Medicine Reminder	SIMPTEK LLP
	Pill Alert Pro - Medication Reminder	LINKLINKS LTD
	Pill Box!	Chris Robert
	Pill Buddy	Vitility International B.V.
	Pill Cabinet	Dima Bart
	Pill Girl	Lab 1618
	Pill in Time	Pavel Zryumov
	Pill me	Rambletta Ltd.
	Pill Monitor	WhiteHedge Technologies
	Pill Oclock	Abel Llopis
	Pill Pro	Les Laboratoires Servier

	Pill Prompter Free	Pinch Studios
	Pill Reminder	Drugs.com
	Pill Reminder	Aplicativos Legais
	Pill Reminder - All in One, Medication Reminder	Sergio Licea
	Pill Reminder - MedRem	Globsis LLC
	Pill Reminder Alarm	Qi Chen
	Pill Reminder Pro	Bahtiyar Polat
	Pill Reminder+	Himanshu Ttariya
	Pill Time	Roman Baev
	Pill Tracker Box	Michael Tram
	Pill!	fausto pagliara
	PillAware	Adam Miller
	Pillbot	Justin Huang
	Pillbox by Tricella	Tricella Inc
	Pillbox	Patryk Mierzejewski
	Pillboxie	Nice Boy LLC
	Pillbug	Neet Technologies LLP
	PillButler	Dharmesh Vaghani
	PillCast	GalileoMD, LLC
	PillDrill	PillDrill, Inc.
	Piller	Albin V. Baaw
	PillManager	Healthnet Limited
	Pillminder	RookSoft Ltd.
	Pillmo	pillmo
	Pillo	Canviz Consulting
	Pillow	Niew Yee Yaw
	PillPal Tracker	MobileSmith, Inc.
	Pills	Alexander Senin
	Pills Genie	Vital Acts Inc.
	Pills Helper	Arnau Asensio
	Pills Memo	Francesco Pellaco
	Pills On Time	Alex Persian
	Pills Ping	carl nohre

	Pills: Medication Reminders	Emma Jones
	PillsAlert	Antonino Arena
	PillTimer	Evan Hildreth
	Pill-Watch	sodacore studios UG
	PillWatcher	Five Flames Mobile
	Pilly!	Orcun Yoruk
	Pilly! 2	Orcun Yoruk
	Pinnula	Domenico Ganino
	Plan-it Med	Play-it Health
	Pocket Pharmacist	Danike, Inc.
	Prescription Reminder	David White
	Propeller Health	Reciprocal Labs
	Rango for Health	Village Care of New York
	ratiopharm pill reminder	ratiopharm GmbH
	Refill Buddy	Cellflare
	Refill Plus	7 Peaks Business Solutions Incorporated
	Remed	Tom Golbach
	Remember My Pills	Universal Computer Consultants
	RheumaTrack RA	axovis GmbH
	RheumaTrack SPA	axovis GmbH
	Rx Buzzer FREE	HiWHo Corporation - DatMobi
	Rx Remind Me	You Can Sleep When You're Done
	Rx Reminder	Hilary Greenleaf
	Rx Tracker	CircleSoft Llc.
	RX2 - Meds and Pill Reminder	Tyrant Ventures
	RX2 - Meds and Pill Reminder lite	Tyrant Ventures
	RxMinder Go	Innovate Wireless Health, Inc.
	rxremind	Softarch Technologies AS
	RxRemind	Robert Hueston
	RxScripts	Michael Webster
	S3 Stroke Survivor Patient Care	Melvyn Weibin

	Sagely	HB + Co
	Sarcoma Storylines	Self Care Catalysts Inc
	Seizure Sync Epilepsy Log	Neutun Labs Inc.
	Senior Assist Free	RedFlag Technologies
	simpills	Ivan Minier
	Simple Medication Log	Morgan Davison
	Simple Pill Tracker	Lukas Valine
	Simplest Pill Reminder - for Girls	Luca Rullo
	Simply for Me	Janssen-Cilag Pty Limited
	Smartinhaler	Adherum (NZ) Limited
	SPAC Drug Adherence	Gitesh Patel
	Start	Iodine Inc.
	Sup	Alex Prushynskyy
	Symple	Symple Health, Inc.
	tabLog	YUKIO NIWA
	Take a Dose	LekSeek Polska Sp. Z o.o.
	Take A Pill	Cappable Limited
	Take a Pill	Yauheni DZEMIASHKEVICH
	Take Me	Tinyyo Limited
	Take That Pill	Mykola Golyash
	TakeMyPill	Klapp & Co
	The Pill	Stephane QUERAUD
	Therapy Reminder	Antonio Gorini
	TimerCap	Rx Timer Cap, LLC
	TookTheMed	R Rezalifar
	Total Epilepsy Recorder	Strategic Vision, Inc.
	track	Adam Pypstra
	Track My Rx	APG Solutions, LLC
	Transplant Hero - Medication Reminder	Transplant Hero LLC
	Unifontis	Herbert Klaeren
	Vethical Pet Care Reminder	VCA Antech Inc.
	Virtual Pillbox	Philippe Charlot
	Visual Pill Box	Melvyn Weibin

	VitaCoach	Science Tribune
	Vitamin Reminder	digiFi LLC
	VitaMind	SagaciousStudio
	viva-app	Aja Technologies LLC
	Voice med reminder lite	Jais Joy
	Wellframe	Wellframe
	Wellnote	DigitalStitch Limited
	Wildwood Pills	Healthnet Limited
	Women's Health Diary	Baskaran Arunasalam

Operating System	App Name	Developer
**Android**	Medisafe Meds and Pill Reminder *(Assessed for quality, analyzed user-reviews- Android App #1)*	Medisafe
	CareZone *(Assessed for quality, analyzed user-reviews-Android App #2)*	CareZone
	MyTherapy Meds and Pill Reminder *(Assessed for quality, analyzed user-reviews-Android App #3)*	MyTherapy
	Med Helper Pill Reminder *(Assessed for quality, analyzed user-reviews-Android App #4)*	Manyeta
	Dosecast - Medication Reminder *(Assessed for quality, analyzed user-reviews-Android App #5)*	Montuno Software, LLC
	My PillBox (Meds&Pill Reminder) *(Assessed for quality, analyzed user-reviews-Android App #6)*	Master B
	Pill Organizer & Reminder *(Assessed for quality, analyzed user-reviews-Android App #7)*	Necessary Software
	Medicine time! *(Assessed for quality, analyzed user-reviews-Android App #8)*	JMSoft Brazil

	AnyTimer Pill Reminder<br>*(Assessed for quality)*	azuzi
	Med Minder - Free<br>*(Assessed for quality)*	Garland Systems Enterprises, LLC
	ADHD Adults	Labs Health Company
	ADHD Kids	Labs Health Company
	AlarMeds alarm pill reminder	Galecode
	AnyTimer 28 Days Pill Reminder	Azuzi
	AppaGrapha Med Reminder	Odin Pharmacy Innovations LLC
	Asthma Tracker	Kantonssptal Baselland
	AsthmaMD	AsthmaMD
	Baby Medication	ASPIRING USER APPS
	Bedsider Reminders	Bedsider
	BioMark Health Epilepsy	BioMark Health Inc.
	Birth Control	NET DIGITALE
	Birth Control Pill Alarm	mamuso
	Biva - Selfcare Wellness Life	Biva
	BonaPill Contraceptive Reminder	ALFA MEDIMEDIA, s.r.o.
	BreatheSmart	CoheroHealth LLC
	Caixa de Remedios	Ambiente-Medicamento
	CareTRx Asthma & COPD Journal	Gecko Health Innovations, Inc.
	CeyHello FREE Pill Reminder	MedSqr LLC
	CF MedCare Reminder App	M-Data
	Chill pill	Kenan Kobic
	CML Today	CML Adovocates Network
	Conran Pharma	Healthnet Ltd
	Contraceptive pill	Joaquin Fernandez
	Contraceptive pill alarm	Michael Asbeck
	Contraceptive pill reminder	TD Incorporation
	Contraceptive pill reminder	FreeAppsTV
	Depression Medication Manager (Early Access)	MoodTools
	DesferApp Meds & Pill Reminder	Appgraid Apps
	Diabetes Metrics	Healthzini, Inc.

DIY Medication Log	ASPIRING USER APPS
Do not forget your Pills	Makeitapp
DOCTL	QualiCure Software Private Limited
Dog Medication Reminder BETA	BriCo84
Dose Direct - Medicine Alert	Omi Studio
Dr. Pills	2soldiers
Dr. Pills Medication Organizer	2soldiers
DrPill	Frank Cusmano
Drug Alarm	EONSOFT
Drug Reminder Free	AppsLand
eMedsMate - Medicine Reminder	Techtree IT Systems Private Limited
Emma Free (pill reminder)	Medicine Men
EpiDiary	Irody, Inc.
Every Dose, Every Day	Centers for Disease Control and Preventation
EZ Pill Reminder	The Power To Go, LLC
Flo Period Tracker	OWHEALTH
Forty-fiveNE Healthcare	Forty-fiveNE Inc.
GameTherapy	Ramanpreet Singh Khinda
GenieMD	GenieMD
Get Pills	Rafal Rzepecki
Glow Ovulation and Fertility	Glow Inc
Headache Log	AR Productions Inc.
HealthyNow	Cerner Corpopration
iChoice Meds	ChoiceMMed America Co.
iEZ Pillbox	Infoengine Technology (Shenzhen) Limited
iMEDTracker for Pills & Meds	Simplified APP Solutions, LLC
INR Diary	Peter Mesotten
I-pill	Mambo S.A.
iPilule Pill Reminder	Kreactive
Lady & Birth Pill Reminder	HiAppo
Lady Pill Alarm	Snow Fox Apps
Lady Pill Reminder	Baviux

	Lady Pill Reminder	Ben Basha
	Lady Pill Reminder	Bouqt
	Lady Pill Widgets	Baviux
	LadysCalendar Pill	GalleryApp
	LadysCalendar Pill Free	GalleryApp
	Med Helper Pro Pill Reminder	Manyeta
	Med Minder - Pill Reminder	Garland Systems Enterprises, LLC
	MedAlarm	MND Apps
	MedAssist QuickList-Meds,Pills	PharMed Solutions, LLC
	MedBeep - Pill Reminder	ConsultaDoctor IT
	Medi Time	Mujtaba
	Medical Alert	H&Y Apps Inc.
	Medicalog for Families	LemonDraft
	Medication	MaxSDev
	Medication Adherence	Right to Care
	Medication Alert	UKinVision
	Medication Assistant	Gra Orlov App
	Medication Control	Igor Fomin
	Medication Dose Log	ASPIRING USER APPS
	Medication Log	Ohad Aloni
	Medication Log (Medicine)	GalleryApp
	Medication Log Free (Medicine)	GalleryApp
	Medication Log Wiz	GalleryApp
	Medication Log Wiz Free	GalleryApp
	Medication Logs	ASPIRING USER APPS
	Medication Manager	Shop An App
	Medication Manager Free	WizGenX Software Solutions Private Ltd
	Medication Minder	Dev Chefs Inc.
	Medication Reminder	Roughi Studio LTD
	Medication Reminder	Martin Pluta
	Medication Reminder	PlanetaAndroid
	Medication Reminder	iHealth Ventures LLC
	Medication Reminder MedRemind+	Innovemind

	Medication Reminder: Medica	IRWAA LLC
	Medication Reminders Widget	Nio
	Medication Time Reminder Alert	iCraft Media Solutions
	Medication Tracker	Mobile Edge
	Medication Vault	Stichting HuKeLou
	Medication& Lady Pill Reminder	AppS Machine
	MedicationPro plugin	MaxSDev
	Medications Control	HIGSOFT Anatol Kotok
	Medicify	Medicify
	medicine - pills time reminder	ah_apps
	Medicine & Pill Reminder	Ashen Code
	Medicine Alarm To Medication	FirstKlass Dev
	Medicine Call - Pill reminder	opoae
	Medicine Dose Reminder	TECNOLOGIA EN SERVICIOS BIOMEDICOS
	Medicine Reminder	Appally
	Medicine Reminder	WebWist
	Medicine Reminder Free	Proven Digital Web Solutions
	Medicine Tracker	Fuzzy Labs
	MediKeep - pill reminder	MediKeep OU
	MediLog(Free)	Maxcom
	MediMate Pill Reminder	MediMate
	MediRem - Medication Reminder	SmartPoint Technologies
	Mediware Medication Reminder	MediWare India
	MedLi Medication Reminder	MooRango
	MedMonitor	Benesalus Technologies LLC
	MedMonitor Data, Pill Reminder	Linkwireless LLC
	MedOClock FREE Pill Reminder	M2C2 Communications Inc.
	Meds & Pills Reminder	Dr. CUCO
	Med's Alarm	Ape Software
	medStrac - Medication Tracker	wanojope
	MedsWithMe	vastuf.com
	MedTimer	Kbiper
	Mega Meds medicine app & game	AppAttic Ltd

	Memo Health	Tinylogics Ltd
	Mind The Pill	Mind The Pill
	Mr. Pillster	Whisper Arts
	My Cycles Period and Ovulation	MedHelp, Inc - Top Health Apps
	My Medication	Tecnologico de Morelia - Alex Amaro
	My Medication	khalid Ali
	My Medication Diary	Baskaran Arunasalam
	My Medication Journal	My Medication Journal
	My Medication Log	paprika
	My Medication Reminders	Medical Health Records
	My Medications	Blancoleon
	My Meds - MedRem	Globsis Information Technologies
	MY PILLBOX Pro (MEDS REMINDER)	Master B
	My Pills	simo-mobile
	My Seizure Diary	Epilesy Foundation
	MyDays X - Period & Ovulation	Christian Albert Mueller
	MyMedManager	Shabbir Kermali
	MyMedRec	ISMP Canada
	MyPersonalNurseL Pill Reminder	Mark Headson
	myPill Birth Control Reminder	Bouqt
	mySymptoms Food Diary	SkyGazer Labs Ltd
	mySymptoms Food Diary (Lite)	SkyGazer Labs Ltd
	nestCARE	nestCARE Inc.
	Neura Meds Reminder	Neura
	NFC Talking Pill Reminder	WhatPills
	Okusurino Jikan	
	One Drop - Diabetes Management	One Drop
	OnTrack Diabetes	Vertical Health
	Ovulation & Period Calendar	Vipos Apps
	PaDiSys	NowPos
	Period & Ovulation Tracker	SMSROBOT LTD
	Period Tracker	Leap Fitness Group

	Period Tracker, My Calendar	Simple Design Ltd.
	Personal Medication Diary	NexusLink Services
	Pet Medication Reminders App	Pet Republic
	Pharma.io Adherence	Water.IO
	Piills Reminder	rahul7star
	Pill	dferreira
	Pill & Meds Reminder	HiAppo
	Pill & Meds Reminder-Med Alert	GO Apps Studio
	Pill Alert	Rooster Lab
	Pill App	Campos
	Pill App Pro	Campos
	Pill Box Control	Jtsamper
	Pill Box!	Chris Andrea Robert
	Pill Buddy	Vitility International B.V.
	Pill Insight	Merck Sharp & Dohme (Malaysia) Sdn Bhd
	Pill Logger - Meds Tracker	Allen Dev Co
	Pill Manager	Lukasz Wroblak
	Pill me	Rambletta Ltd
	Pill Medication Reminders App	IGRI Studio
	Pill Planners	Arena Phone BD Ltd.
	Pill Pro	LES LABORATOIRES SERVIER
	Pill Reminder	Aplicativos Legais - LTDA
	Pill Reminder	Andrea Canevari
	Pill reminder	Webster, spol. s r.o.
	Pill Reminder	Arthi-soft Mobile Apps
	Pill Reminder	Medsave
	Pill reminder	Joao Sardinha
	Pill Reminder	omnihealth
	Pill Reminder	Pepe Argento
	Pill Reminder	BrianCo
	Pill Reminder - Medicine Timer	RDCT
	Pill Reminder / Birth Control	SMSROBOT LTD
	Pill Reminder AdFree Plus	RDCT

	pill reminder with voice	MYFREEAPPS.DE
	Pill Time	Karam Alem
	Pill Watcher	Five Flames Mobile
	PillManager	Healthnet Ltd
	PillMe: the pill scheduler app	nathan nakhjavani
	PillOrga | Medicine Reminder	Trendaty
	Pillreminder	Bytetex
	PillRx - Pill Reminder	appyown
	Pills	Teun Vos
	Pills Alarm	Pills Alarm
	Pills Every Hour or Two or 3..	David Gray
	Pills Notifier	GolanHershko
	Pills on the Go	App Singularity
	Pills on the Go - Free	App Singularity
	Pills Reminder	CS IT Park
	Pills Reminder	Appliccon Sp. Z o.o.
	Pills Reminder - Health Helper	S Health Helper
	Pills Reminder Free - MedAlarm	S Health Helper
	Pills Reminder PRO	Appliccon Sp. Z o.o.
	Pills'Box	Softetic
	Pills'Box FREE	Softetic
	PocketNurse - Pill Reminder	Instait
	ratiopharm pill reminder	ratiopharm GmbH
	Remeds: medication reminder	Montesoft
	Reminder medication alert	thewindoftechnology
	Rx Medicine Reminder	Fives Desk
	RX Pal Family Pill Minder Free	Neca Soft
	RX Pal Family Pill Reminder	Neca Soft
	RX Pal Medication Reminder	Neca Soft
	RxManager-Free	A5Software
	RxMinder GO	Innovate Wireless Health, Inc.
	Rxremind Meds & Pill Reminder	Softarch Technologies AS
	RxTime Pill Reminder	CJ Riverstone LLC

	RxTime Pro Pill Reminder	CJ Riverstone LLC
	Skin Medication Monitor	SHUEWE
	Smart Medication Tracker	UrFamily Pte. Ltd.
	Smart Pill Reminder	ApnaCare India Private Limited
	Smart Pill Station	ASITEQ
	SmartMED	Imaginauts Corp.
	Start	Iodine, Inc.
	Take a pills	Mikhail Voronin
	Take The Pill	Marcelo Alvefs Rezende
	Take your medications	Issam KHADIRI
	Take Your Pills	Tandem Tehran
	Take your pills!	difer
	TB Management System	Embryyo Technologies Pvt Ltd
	ThalCare	Jagriti Innovations
	The Pill	Stephane QUERAUD
	Timely Meds	SLIMS CAPITAL LP
	Track My Rx	APG Solutions, LLC
	Transplant Hero	Transplant Hero LLC
	TreatUp med agenda, pill remind	Franc Pharma
	Vesta Pills	2soldiers
	Virtual Pill Box	Avinash Kulkarni
	Visual Pill Box	Melvyn Zhang Weibin
	Visual Pill Reminders	Innotion Technologies
	Wildwood Pills	Healthnet Ltd
	WomanLog Calendar	Pro Active App
	WomanLog Pro Calendar	Pro Active App
